# Family History of Breast Cancer and Breast Cancer Risk between Malays Ethnicity in Malaysia and Indonesia: A Meta-Analysis

**Published:** 2019-02

**Authors:** Ricvan Dana NINDREA, Teguh ARYANDONO, Lutfan LAZUARDI, Iwan DWIPRAHASTO

**Affiliations:** 1. Doctoral Program, Faculty of Medicine, Public Health and Nursing, Universitas Gadjah Mada, Yogyakarta, Indonesia; 2. Department of Public Health, Faculty of Medicine, Universitas Andalas, Padang, Indonesia; 3. Department of Surgery, Faculty of Medicine, Public Health and Nursing, Universitas Gadjah Mada, Yogyakarta, Indonesia; 4. Department of Health Policy and Management, Faculty of Medicine, Public Health and Nursing, Universitas Gadjah Mada, Yogyakarta, Indonesia; 5. Department of Pharmacology and Therapy, Faculty of Medicine, Public Health and Nursing, Universitas Gadjah Mada, Yogyakarta, Indonesia

**Keywords:** Breast neoplasms, Family history, Malaysia, Indonesia

## Abstract

**Background:**

Breast cancer is the most common cancer type in women not only in world but also in Malays ethnicity between Malaysia and Indonesia. Breast cancer has varying incidence in every country, but genetic factor by family history influence the incidence of breast cancer. This systematic review and meta-analysis was performed to determine family history of breast cancer and breast cancer risk between Malays ethnicity in Malaysia and Indonesia.

**Methods:**

This meta-analysis was conducted on published research articles on family history of breast cancer and breast cancer risk between Malays ethnicity in Malaysia and Indonesia published between Jan 1999 and Jul 2018 in the online article databases of PubMed, ProQuest and EBSCO. Pooled odds ratios (OR) were calculated with fixed and random-effect models. Publication bias was visually evaluated by using funnel plots and statistically assessed through Egger’s and Begg’s tests. Data were processed using Review Manager 5.3 (RevMan 5.3) and Stata version 14.2 (Stata Corporation).

**Results:**

We reviewed 1123 articles. There are 10 studies with number of samples 4511 conducted a systematic review and continued with Meta-analysis of relevant data. The results showed significant association between family history of breast cancer with breast cancer risk in Malays ethnicity in Malaysia and Indonesia (OR = 3.34 [95% CI 2.68–4.15, *P*<0.00001]). There was not significant publication bias for studies included in family history of breast cancer and breast cancer risk in Malays ethnicity in Malaysia and Indonesia.

**Conclusion:**

This analysis confirmed the association of family history of breast cancer and breast cancer risk between Malays ethnicity in Malaysia and Indonesia.

## Introduction

Breast cancer ranks first of all cancer disease in women encountered worldwide (1). An estimated 23% or 1,383,500 new cases a year and 14% or 458,400 cases will end in death (2). Breast cancer is the most common cancer type in women not only in world but also in Malays ethnicity between Malaysia and Indonesia. Cancer data and risk factors from Malaysia and Indonesia are poor (3).

Breast cancer is a disease caused by multifactorial: age, genetic and inherited, estrogen replacement, oral contraceptive, carcinogen exposure, alcohol consumption, high fat consumption, and smoking. Breast cancer incidence differs in every country (3–5). North America and West Europe have higher incidence than Asia. This fact shows that not only breast cancer has varying incidence in every country, but genetic factor by family history influence the incidence of breast cancer. Previous study define risk levels associated with varying degrees of positive family history.

The degree of risk was a function of the type of relative affected (first or second degree), the age at which the relative developed cancer, and the number of relatives affected. Compared to individuals with no family history of breast cancer (6).

Subjects with a family history of first-degree relatives including sisters, mothers or children with breast cancer or distant relatives, grandmothers, grandchildren, aunts or nephews, who had breast cancer showed higher risk (OR=2.95 and OR=2.84) compared with the results of the meta-analysis of OR = 1.5–2.1 (6). Family history have risk factors for breast cancer compared to women with no family history with the results of the meta-analysis in Southeast Asia (3).

Increasing comprehensive knowledge of a ***family history*** of breast cancer and awareness of breast cancer risk could facilitate its early detection. It can be more effectively treated in earlier stage than when clinical signs and symptoms present, justifying early detection efforts. Through measurement of breast cancer risk, it can be seen whether a person has a safety risk to breast cancer, adequate for breast cancer prevention or harmful to the occurrence of breast cancer (7,8). Therefore, we aimed to determine family history of breast cancer and breast cancer risk between Malays ethnicity in Malaysia and Indonesia with some research through the Meta-analysis study so that the conclusion drawn have stronger strength.

## Materials and Methods

### Study design and research sample

This study was quantitative research with meta-analysis study design. The meta-analysis followed the preferred reporting items for systematic reviews and meta-analysis (PRISMA) statement (9). Meta-analysis was used to figure family history of breast cancer and breast cancer risk in Malays ethnicity in Malaysia and Indonesia. The research samples were published research articles published between Jan 1999 and Jul 2018 in online article databases of PubMed, ProQuest, and EBSCO.

### Operational definitions

The variables of this study included independent variable is family history of breast cancer, and dependent variable is breast cancer in Malays ethnicity in Malaysia and Indonesia.

### Research procedure

**Fig. 1:**
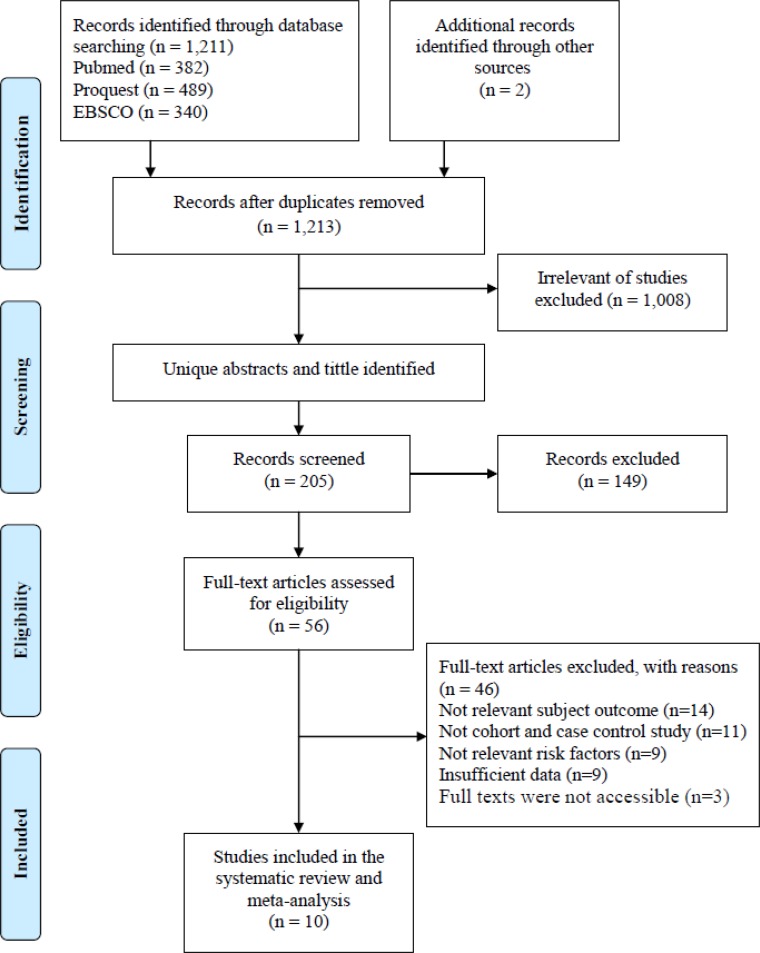
Flow diagram research procedure

This study was conducted by collecting data through the identification of published research articles on family history of breast cancer and breast cancer risk in Malays ethnicity in Malaysia and Indonesia in online article databases of PubMed, ProQuest and EBSCO (Fig. 1). Identification of 1,213 articles, done by review through the title of the articles, continued by reviewing the abstract, and then the full-text form. The article was excluded if: (a) not relevant subject outcome, (b) not case-control and cohort study (c) the information provided in the results were insufficient for data extraction.

### Data collection technique

The data collection was done through online search. The research was limited to English language articles. The article type was limited to journal articles. The research subject was limited to research with human subject. The abstract of articles with potentially relevant titles was reviewed, while the irrelevant articles were excluded.

Furthermore, articles that have potentially relevant abstracts will be reviewed in full-text, while the irrelevant articles were excluded. The inclusion criteria of this study sample were research on family history of breast cancer and breast cancer risk in Malays ethnicity in Malaysia and Indonesia with case-control and cohort study. Exclusion criteria were: the research was not available in full text form and when these criteria were not satisfied or if the provided information was insufficient for data extraction. The following data were obtained from each article: first author’s name and year of publication, region, type of study and number of sample.

Two independent investigators carefully extracted information from all studies that satisfied the inclusion criteria in accordance with a standardized protocol. Disagreements were resolved by three other investigators. Quality assessment was conducted using Newcastle–Ottawa Quality Assessment Scale (NOS). The papers with a total score of 0–3, 4–6, and 7–9 points were specified as the poor, moderate, and high quality (10).

### Data analysis

The analysis held to get the value of pooled odds ratio which is the combined odds ratio value from the research. Results were pooled using odds ratio with corresponding 95% confidence intervals (CIs). Significant heterogeneity was indicated by *I*^2^>50% because these tests presented minimal statistical power in cases with few studies and small sample sizes. A random effect model was used when significant heterogeneity was observed; otherwise, a fixed effect model was utilized. Data were analyzed by using Review Manager 5.3 (RevMan 5.3).

Publication bias was visually evaluated by using funnel plots and statistically assessed through Egger’s and Begg’s tests. Meta-analysis was carried out in Stata version 14.2 (Stata Corporation). A two-tailed *P*-value of <0.05 was considered statistically significant.

## Results

**Fig. 2:**
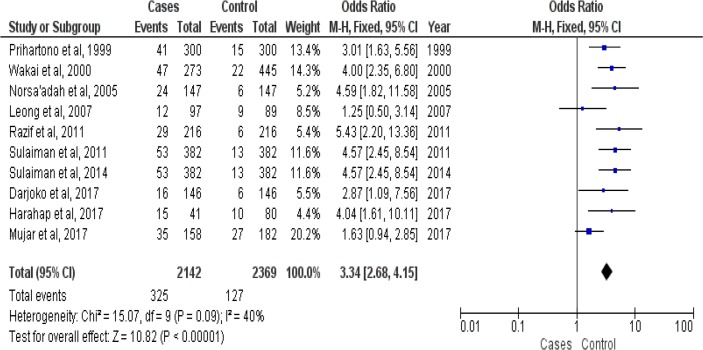
Forest plots family history of breast cancer and breast cancer risk between Malays ethnicity in Malaysia and Indonesia

**Fig. 3:**
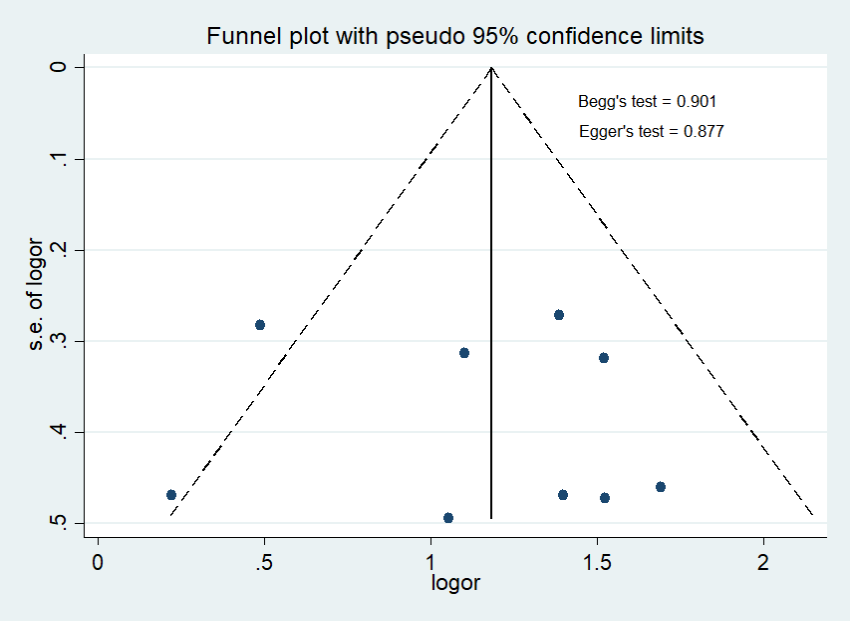
Funnel plot to examine publication bias for family history of breast cancer and breast cancer risk between Malays ethnicity in Malaysia and Indonesia

The selection of studies was conducted to obtain 10 studies with number of samples 4511 related to family history of breast cancer and breast cancer risk in Malays ethnicity in Malaysia and Indonesia (Table 1). Figure 2 shows meta-analysis of family history of breast cancer and breast cancer risk in Malays ethnicity in Malaysia and Indonesia (OR = 3.34 [95% CI 2.68–4.15, *P*<0.00001]). Heterogeneity among studies for family history of breast cancer and breast cancer risk in Malays ethnicity in Malaysia and Indonesia (*P*_heterogeneity_ =0.09; I^2^=40%) had a variation of homogeneous research for the occurrence of breast cancer. Funnel plots for identify publication bias among studies family history of breast cancer and breast cancer risk in Malays ethnicity in Malaysia and Indonesia (Fig. 3). There was not significant publication bias for studies included in family history of breast cancer and breast cancer risk in Malays ethnicity in Malaysia and Indonesia, Egger’s test (*P*=0.877) and Begg’s test (*P*=0.901).

**Table 1:** Systematic review of family history of breast cancer and breast cancer risk between Malays ethnicity in Malaysia and Indonesia

***First Author, Year***	***Region***	***Type of Study***	***Number of Samples***		***NOS***
			***Cases***	***Control***	
Prihartono et al ( 11 )	Indonesia	Case Control	300	300	8
Wakai et al ( 12 )	Indonesia	Case Control	273	445	8
Norsa’adah et al (13)	Malaysia	Case Control	147	147	7
Leong et al (14)	Malaysia	Case Control	97	89	6
Razif et al (15)	Malaysia	Case Control	216	216	7
Sulaiman et al (16)	Malaysia	Case Control	382	382	7
Sulaiman et al (17)	Malaysia	Case Control	382	382	7
Darjoko et al (18)	Indonesia	Case Control	146	146	7
Harahap et al (19)	Indonesia	Cohort	41	80	6
Mujar et al (20)	Indonesia	Case Control	158	182	7
Total			2142	2369	

Abbreviation: NOS, Newcastle–Ottawa Quality Assessment Scale

## Discussion

Our result showed significant association between family history of breast cancer with breast cancer risk in Malays ethnicity in Malaysia and Indonesia (OR=3.34 [95% CI 2.68–4.15, *P*<0.00001]). There was no significant publication bias for studies included in family history of breast cancer and breast cancer risk in Malays ethnicity in Malaysia and Indonesia. This study revealed the similarity that subjects with a family history of breast cancer had a significantly higher risk of breast cancer than those not have a family history, consistent with other studies (6, 21). Subjects with a family history of first-degree relatives including sisters, mothers or children with breast cancer or distant relatives, grandmothers, grandchildren, aunts or nephews, who had breast cancer showed higher risk (OR = 2.95 and OR = 2.84) compared with the results of the meta-analysis of OR = 1.5–2.1 (6).

Women with a positive history of breast cancer were more likely to adhere to screening guidelines (22, 23), however, women with breast cancer who have a positive family history do not appear to present with earlier stages or smaller tumours (24). However, in general, study breast cancer risk, strong evidence exists for increased risk in individuals having a family of breast cancer (6). This thing causes the percentage of breast cancer patients who came to treatment at an advanced stage showed that the lack of early detection behavior performed by women, as well as the lack of awareness of women and understanding of breast cancer primarily in women who have family history of breast cancer risk factors for breast cancer and early detection, less applied so that most women come in breast cancer conditions at an advanced stage (3,4).

The family history with promoter methylation of BRCA1 gene is significantly higher than previously reported from other population such as Taiwanese present in 56%, Thailand 24.6%, India 45% but present similar highest status with Vietnamese, 82,1% and France, 89,1% (25–29). Family history have risk factors for breast cancer compared to women with no family history with the results of the meta-analysis in Southeast Asia (3). Our study supports previous studies, which also relates to breast cancer (30, 31).

There were a few limitations in this meta-analysis. First, three studies seemed potentially eligible to be included in this meta-analysis but the full texts were not accessible. This issue may raise the possibility of selection bias. Second, the number of cases sample in one study is relatively small (19), which can reduce the statistical power.

This analysis confirmed the association of family history of breast cancer and breast cancer risk in Malays ethnicity in Malaysia and Indonesia. The results of this study recommend women with a family history of breast cancer should be counseled and educated about the risk of breast cancer, and it is recommended to make early detection of breast cancer. Family history of breast cancer in the close relatives was related to an increased risk of breast cancer, which suggests the existence of genetic or environmental factors that are shared among relatives and modifies the risk of breast cancer. The family history of breast cancer needs additional counseling, psychosocial support, and screening breast cancer risk to ensure their overall outcomes of breast cancer are optimized. For health workers in order to provide feedback to the community through counseling, especially mothers about the importance of knowing the risk factors that affect the incidence of breast cancer and to detect it early with Breast Self-Examination (BSE) that breast cancer can be detected early because of breast cancer is discovered in stage early can still be cured, so that the incidence of cancer is no longer increasing.

## Conclusion

This analysis confirmed the association of family history of breast cancer and breast cancer risk between Malays ethnicity in Malaysia and Indonesia

## Ethical considerations

Ethical issues (Including plagiarism, informed consent, misconduct, data fabrication and/or falsification, double publication and/or submission, redundancy, etc.) have been completely observed by the authors.
